# SLP-76 Sterile α Motif (SAM) and Individual H5 α Helix Mediate Oligomer Formation for Microclusters and T-cell Activation*

**DOI:** 10.1074/jbc.M112.424846

**Published:** 2013-08-09

**Authors:** Hebin Liu, Youg Raj Thaker, Loren Stagg, Helga Schneider, John E. Ladbury, Christopher E. Rudd

**Affiliations:** From the ‡ Cell Signalling Section, Department of Pathology, University of Cambridge, Tennis Court Road, Cambridge CB2 1QP, United Kingdom,; the § Department of Biological Sciences, Xi'an Jiaotong-Liverpool University, 111 Renai Road, Suzhou Industrial Park, Jiangsu Province 215123, China, and; the ¶ Department of Biochemistry and Molecular Biology, The University of Texas MD Anderson Cancer Center, Houston, Texas 77030

**Keywords:** Adaptor Proteins, Biophysics, Immunology, Protein Complexes, Signal Transduction, T-cell, SAM Domain, SLP-76

## Abstract

Despite the importance of the immune adaptor SLP-76 in T-cell immunity, it has been unclear whether SLP-76 directly self-associates to form higher order oligomers for T-cell activation. In this study, we show that SLP-76 self-associates in response to T-cell receptor ligation as mediated by the N-terminal sterile α motif (SAM) domain. SLP-76 co-precipitated alternately tagged SLP-76 in response to anti-CD3 ligation. Dynamic light scattering and fluorescent microscale thermophoresis of the isolated SAM domain (residues 1–78) revealed evidence of dimers and tetramers. Consistently, deletion of the SAM region eliminated SLP-76 co-precipitation of itself, concurrent with a loss of microcluster formation, nuclear factor of activated T-cells (NFAT) transcription, and interleukin-2 production in Jurkat or primary T-cells. Furthermore, the H5 α helix within the SAM domain contributed to self-association. Retention of H5 in the absence of H1–4 sufficed to support SLP-76 self-association with smaller microclusters that nevertheless enhanced anti-CD3-driven AP1/NFAT transcription and IL-2 production. By contrast, deletion of the H5 α helix impaired self-association and anti-CD3 induced AP1/NFAT transcription. Our data identified for the first time a role for the SAM domain in mediating SLP-76 self-association for T-cell function.

## Teaser

**Background:** SLP-76 possesses an N-terminal sterile α motif (SAM) domain of unknown function.

**Results:** SLP-76 SAM and its isolated H5 domain self-associates for microclusters and NFAT transcription.

**Conclusion:** SLP-76 self-associates in response to T-cell receptor (TCR) ligation as mediated by the SAM domain.

**Significance:** SAM-mediated SLP-76 dimerization is crucial to understanding how SLP-76 forms complexes for T-cell activation.

## Introduction

T-cell activation and effecter functions are initiated by plasma membrane proximal protein-tyrosine kinases and their phosphorylation of an array of downstream substrates (1–3). Among these substrates are adaptor proteins and molecular scaffolds that mediate the formation of multimeric complexes that integrate signals for various functions (2, 4, 5). Src homology 2 (SH2) domain-containing leukocyte protein of 76 kDa (SLP-76;^3^ also known as LCP2, lymphocyte cytosolic protein) in T-cells is an adaptor protein that is needed for thymic differentiation and mature T-cell function (5, 6). Its loss impaired the activation of phospholipase Cγ1, calcium mobilization, adhesion, and thymic differentiation (7–11).

Structurally, SLP-76 is comprised of an N-terminal sterile α motif (SAM), three tyrosine motifs (YESP, YESP, and YEPP), a central proline-rich region and a carboxyl-terminal SH2 domain (6). Residues Tyr-113, Tyr-128, and Tyr-145 are phosphorylated by ZAP-70 (12, 13), whereas Tyr-113 and Tyr-128 bind to the guanine nucleotide exchange factor Vav1 and the adaptor, non-catalytic region of tyrosine kinase adaptor protein 1 (Nck) (6). SLP-76 also binds to Tec kinases, resting lymphocyte kinase (14), and inducible tyrosine kinase (15). The latter binding depends on Tyr-145 (16, 17). In contrast, the proline-rich region of SLP-76 binds to Gads (Grb2-related adapter protein) and phospholipase γ1 (18–21). Gads binds via its SH3 domain to a non-canonical RSTK motif (22), whereas the phospholipase Cγ1 SH3 domain binds to the proline-rich region (23–26). At the C-terminal end of SLP-76, the SH2 domain binds to ADAP (adhesion and degranulation-promoting adapter protein) (27, 28) and the hematopoietic progenitor kinase-1 (29–31). ADAP activates lymphocyte function-associated antigen 1 (LFA-1) via SKAP1 (Src kinase-associated phosphoprotein 1) and its requirement in the formation of the RapL-Rap1 complex (4, 32, 33). SLP-76 also forms microclusters for signaling (34–36) and can exert feedback control on ZAP-70 clustering (37). SLP-76 clusters also interact with subsynaptic LAT (linker for activation of T-cells) clusters from intracellular vesicular compartments (38, 39).

SAM domains are found in numerous surface receptors, signaling proteins, and transcription factors (40). To date, <25% of SAM domains have been reported, or predicted, to form dimers/oligomers either between themselves or with other proteins (41). Examples include transcription factors such as the ETS transcription factor TEL, and polyhomeotic, as well as cell surface receptors ephrin B (EphB) and LAR (leukocyte common antigen-related) (42–45). Their versatility in binding has implicated them in an array of biological processes that includes signal transduction, protein translation, and gene transcription (46, 47). Structurally, the SAM regions are generally comprised of multiple α helices (H1–H5). The crystal structure of the self-associating EphB2 SAM domain has shown that presence of two binding interfaces, one formed by adjacent monomer exchange of amino-terminal peptides that insert into a hydrophobic groove on each neighbor and a second composed of the carboxyl-terminal H5 helix and a nearby loop (44, 48). The SLP-76 SAM region is comprised of residues 1–78 with five conserved α helices (H1–5) (6, 48). Previous work has shown that the partial loss (*i.e.* residues 12–78) of the SLP-76 SAM region can impair positive and negative thymic selection (49).

Despite the importance of SLP-76, it has been unclear whether the adaptor can directly self-associate in response to T-cell receptor ligation and whether this event is needed for the activation of T-cells. Although complexes comprised of SLP-76 associated with adaptors such as Nck and Vav-1 have been described (50), the direct binding of SLP-76 to SLP-76 has not been reported. Here, we report that anti-CD3 induces SLP-76 self-association mediated by the SAM domain, and this event was needed for SLP-76 microcluster formation and T-cell activation. Furthermore, different regions in the SAM domain contributed to this self-association with the H5 helix alone supporting co-precipitation of SLP-76 at reduced levels, smaller microclusters, and enhanced T-cell activation. Our data identified for the first time that anti-CD3 ligation induces SLP-76 self-association as mediated by its N-terminal SAM domain.

## EXPERIMENTAL PROCEDURES

### 

#### 

##### Cell Culture, Reagents, and Expression Vectors

SLP-76-deficient Jurkat J14 T-cells (gift from A. Weiss, University of California, San Francisco) were cultured as described (51). CD4^+^ mouse DO11.10 T-cells were isolated using Dynabeads (Dynal Biotech ASA, Oslo, Norway), and human T-cells by centrifugation of Ficoll Hypaque (52). Monoclonal antibodies used included anti-human CD3 (OKT3), anti-mouse CD3 2C11 (American Type Culture Collection), anti-SLP-76 (BioXcell, West Lebanon, NH), anti-HIS (Cell Signaling, Danvers, MA), and anti-ADAP and GADS (Upstate Biotechnology, Lake Placid, NY). SLP-76-EYFP was constructed by subcloning SLP-76 cDNA into the XhoI/BamHI sites of pEYFP-N1 vector (Clontech, Madison, WI). The dN57 mutant was generated by replacing the full-length SLP-76 with PCR-amplified cDNA coding 58 to 533 amino acids into SpeI/BamHI sites of SLP-76-EYFP plasmid. The SLP-76-EYFP dN78 mutant was generated by replacing with PCR amplified cDNAs coding 79 to 533 amino acids. C-terminally His_6_-tagged SLP-76 and dN57 and dN78 were cloned into the XhoI/Kpn1 sites of SRα vector. The SLP-76 mutants lacking the H5 domain (ΔH5), tagged with His_6_ or EYFP, respectively, were generated by site-directed mutagenesis using primers 5′-gacatccagaagttcaggagcatcttcacacgc-3′ and 5′-gcgtgtgaagatgctcctgaacttctggatgtc-3′. All of the mutations in SLP-76 mutants were confirmed by DNA sequencing. Jurkat J14 T-cells were transfected by microporation (Digital BioTechnology), using a single pulse of 30 ms at 1410 V, and mouse DC27.10 cells with 2 pulses of 20 ms at 1400 V. Mouse CD4 primary T-cells and human peripheral T-cells were transfected by Nucleofector (Lonza, Cologne, Germany). [^3^H]Thymidine incorporation was conducted as described (53). For luciferase assays, T-cells were transfected with 10 μg of NFAT-luc and 5–10 μg of expression vector followed anti-CD3 ligation and measurement of luciferase activity (52).

##### Confocal Imaging

Live cell imaging on polylysine (Sigma) and anti-CD3-treated cover slides (LabTek, Rochester, NY) was conducted as described (36–38, 54). Cells were imaged by resonance scanning confocal microscopy (TCS SP5 RS, Leica, Heidelberg, Germany) using excitation wavelengths of 514 nm (for EYFP) and 594 nm (for mCherry). Images were processed with Leica confocal software (Leica Microsytems), Volocity (Improvision), and ImageJ software (National Institutes of Health).

##### Recombinant SLP-76 N-terminal SAM Domain Protein Purification

The cDNA encoding the SLP-76 N-terminal SAM domains from 1–78 (H1–5) and 1–61 (H1–4) amino acids were subcloned into NdeI/BamHI sites of pET-20b(+) (Novagen, Madison, WI) and used to transform *Escherichia coli* BL21(DE3) cells. The soluble fractions, containing the expressed recombinant proteins, were then purified by Ni^2+^ column (Qiagen, Hilden, Germany). Immunoprecipitation and blotting was conducted as described (32, 33).

##### Fluorescence Microscale Therophoresis (MST)

MST experiments were performed using a Monolith NT.115 instrument (NanoTemper) as reviewed (55). Temperature was controlled at 25 °C in the following buffer: 10 mm HEPES, pH 7.5, 500 mm NaCl, 0.5 mm tris(2-carboxyethyl)phosphine, and 0.05% Tween 20. Standard glass capillaries were used. Fluorescence labeling of SLP-76 SAM domain was performed using primary amide coupling of NT-647 dye (NanoTemper) using the manufacturer's instructions. A labeling efficiency of one label per one protein molecule was verified by spectrophotometric analysis using the following molar extinction coefficients (Σ_280_ SLP-76 SAM = 9,970 m^−1^ cm^−1^; Σ_650_ NT-647 = 250,000 m^−1^ cm^−1^). Individual titrations of 10 nm NT-647 labeled SLP-76 SAM domain and unlabeled SLP-76 SAM domain (0–90.5 μm) were made via 1:1 dilution from stock protein. The reported monomer-dimer *K_D_* value was calculated using Origin software from the averages of two separate experimental setups and 11 full titration series, including a range of thermal gradients (from ∼3–12 °C).

##### Circular Dichroism (CD)

The native far-UV CD spectrum of SLP-76 SAM domain was obtained using a Jasco J-810 instrument with temperature control (25 °C; Julabo AWC 100) in the following buffer: 10 mm HEPES, pH 7.5, 150 mm NaCl, and 0.5 mm tris(2-carboxyethyl)phosphine. The reported spectrum is the average of four individual spectra using a 1-mm path length cuvette.

##### Dynamic Light Scattering (DLS)

DLS experiments were performed using a temperature-controlled (25 °C) DynaPro instrument (Protein Solutions). Raw data were analyzed using manufacturer provided Dynamics software (version 6; Wyatt Technologies), and each data point was the result of at least three averages of 20 individual scans. An estimation of SLP-76 SAM domain monomer hydrodynamic radius was performed using the deposited Protein Data Bank structure of the NMR solution structure of SLP-76 SAM Domain (Protein Data Bank code 2EAP), including flanking residues to mimic the expression construct and submitted to HYDROPRO (56). The approximate dimer (and tetramer) hydrodynamic radius was calculated using HYDROPRO with a dimer model constructed by GRAMM-X (57, 58) using the 2EAP structure.

## RESULTS

### 

#### 

##### SAM Domain of SLP-76 Mediates Dimer/Oligomer Formation

**FIGURE 1.**
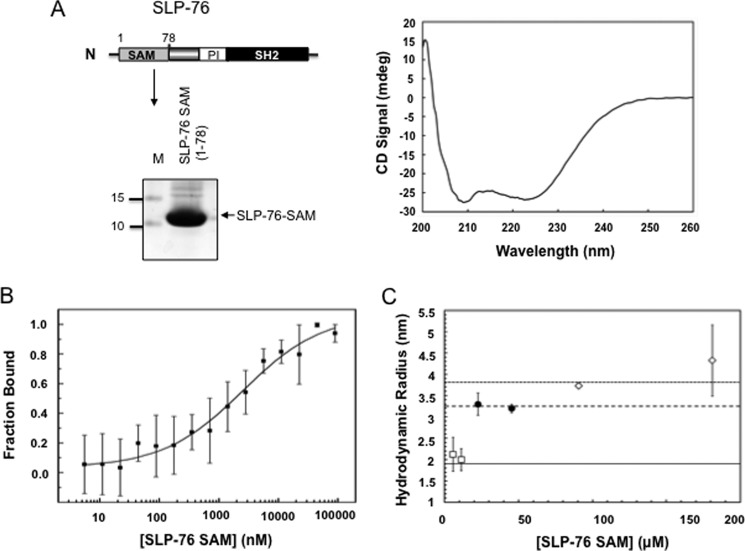
**MST and DLS analysis of isolated SLP-76 SAM protein.** *A*, circular dichroism spectrum of SLP-76. The cDNA encoding the SLP-76 N-terminal SAM domains from 1–78 (H1–5) amino acids were subcloned and used to transform *E. coli* BL21(DE3) cells as described under “Experimental Procedures.” The soluble fractions, containing the expressed recombinant proteins, were then purified by Ni^2+^ column affinity chromatography and examined by CD and showed a characteristic α helical secondary structural content. *Left panel*: SDS-PAGE image of purified protein; *N*, amino terminus; *M*, Molecular weight; *right panel*: CD spectrum of the isolated protein. *B*, MST of the purified recombinant SAM domain. SLP-76 SAM domain was titrated with increasing amounts of unlabeled SLP-76 SAM domain as described under “Experimental Procedures.” The calculated monomer-dimer *K_D_* is 2.5 ± 0.9 μm. *C*, DLS data showing increase in size of hydrodynamic radius of SLP-76 SAM domain with increase in protein concentration. Estimated hydrodynamic radii for SLP-76 SAM domain monomer (*solid line*), dimer (*large dashed line*), and tetramer (*small dashed line*) were determined using the programs HYDROPRO and GRAMM-X as detailed under “Experimental Procedures.” Experimental data correlating to the calculated size of monomer (*open squares*), dimer (*closed circles*), and tetramer (*open diamonds*) are shown with experimental S.D.

A minority of SAM domains have been reported to undergo complex formation with themselves or other proteins (41, 42). Given the importance of complexes to signal transduction, we assessed whether SLP-76 could self-associate and whether the SLP-76 SAM domain could form oligomers. We therefore first examined the *in vitro* binding of a recombinant, purified SLP-76 SAM domain (amino acids 1–78). HIS-tagged human protein corresponding to the SLP-76 SAM domain region (1–78 amino acids) was expressed in *E. coli* followed by purification using Ni^2+^ affinity column chromatography. This procedure yielded a single major protein at ∼10 kDa. (Fig. 1*A*, *left panel*). Purified SLP-76 SAM domain was then analyzed by far-UV CD to verify the α helical secondary content that is typical of natively folded SAM domains (Fig. 1*A*, *right panel*). Two CD bands observed at ∼210 and 225 nm are characteristic of high helical content protein structures, as described for other SAM domains (59). MST performed by titrating increasing concentrations of unmodified SLP-76 SAM domain into fluorescently labeled SLP-76 SAM domain suggested a monomer-dimer *K_D_* of 2.5 ± 0.9 μm (Fig. 1*B*). In addition, DLS corroborated the oligomerization of the SLP-76 SAM domain (Fig. 1*C*). Analysis of DLS data (see “Experimental Procedures”) indicated that the SLP-76 SAM domain formed dimers as well as higher order oligomers. Estimated hydrodynamic radii for SLP-76 SAM domain are shown as a monomer (*solid line*), dimer (*large dashed line*), and tetramer (*small dashed line*) as determined by the programs HYDROPRO and GRAMM-X. The *R*_H_ showed a monomer radius of ∼2 nm that increased to a 3-nm species at a concentration transition of 10 to 20 μm that correlated with a size shift from 10 to 20 kDa. Tetramers were also observed at concentrations >50 μm as well as possible higher order complexes >150 μm. These findings indicated that the SLP-76 SAM domain is capable of self-associating in the formation of dimers and higher order oligomers in solution.

##### SLP-76 Self-associates in Response to CD3 Ligation

**FIGURE 2.**
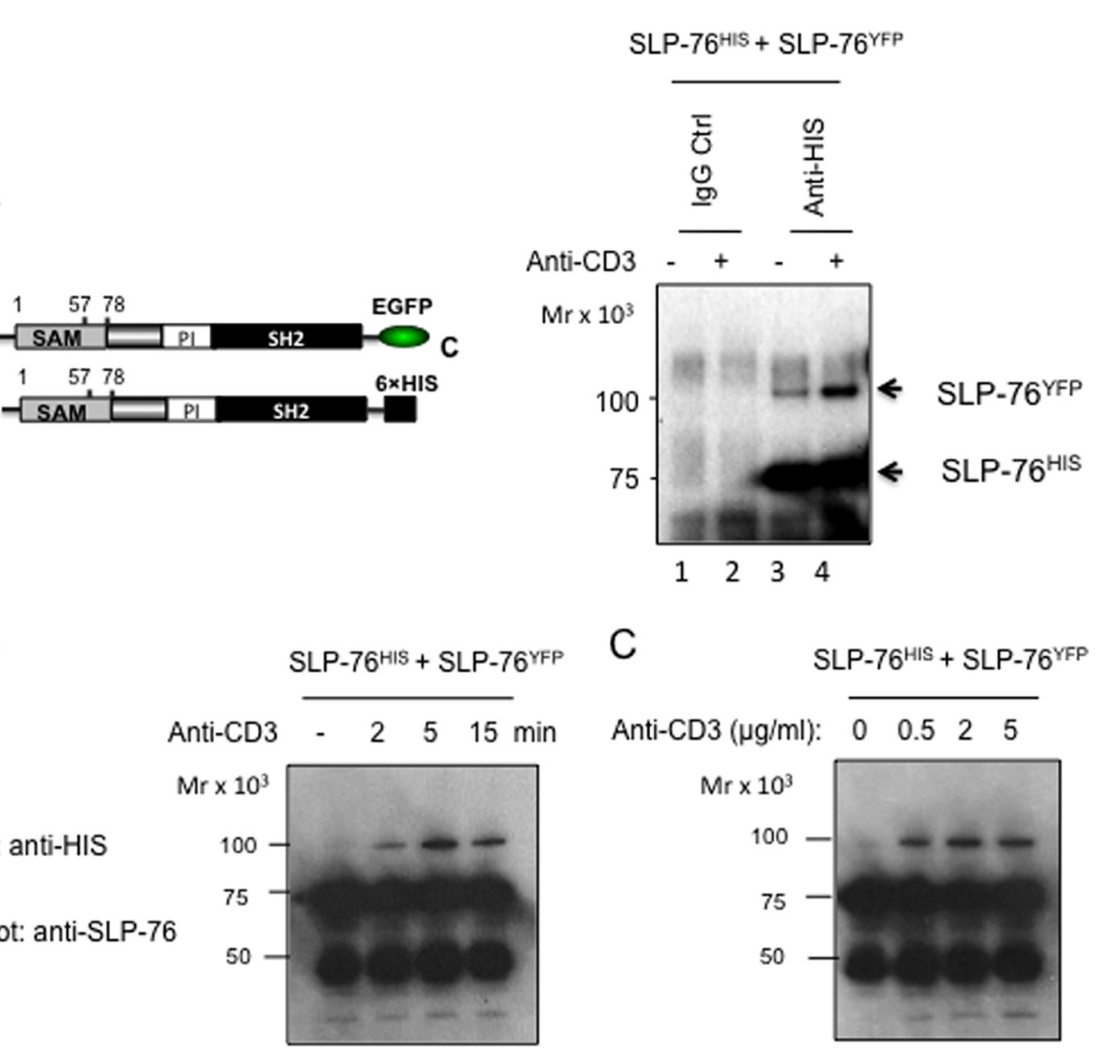
**SLP-76 associates with SLP-76 in response to anti-CD3 stimulation.** *A*, SLP-76 deficient J14 Jurkat T-cells were co-transfected with two tagged versions of SLP-76, one with a EYFP and another with a His_6_ tag (*n* = 3). Twenty-four hours post-transfection, cells were stimulated with anti-CD3 followed by anti-His precipitation of His-tagged SLP-76 and blotting with anti-SLP-76. *Lanes 1* and *2*, anti-IgG control; *lanes 3* and *4*, anti-His precipitation. *lanes 1* and *3*, isotype anti-IgG control; *lanes 2* and *4*, anti-CD3 ligated. The *arrows* indicate the EYFP-SLP-76 (*higher band*) and His_6_-SLP-76 (*lower bands*). *B*, time course of stimulation on SLP-76 binding to SLP-76. *Lane 1*, anti-IgG control; *lane 2*, 2 min; *lane 3*, 5min; *lane 4*, 15 min post-ligation. *C*, anti-CD3 titration stimulation on SLP-76 binding to SLP-76. *Lane 1*, isotype IgG control; *lane 2*, 0.5 μg/ml; *lane 3*, 2 μg/ml; *lane 4*, 5 μg/ml (*n* = 3). *N*, N terminus; *C*, C terminus.

**FIGURE 3.**
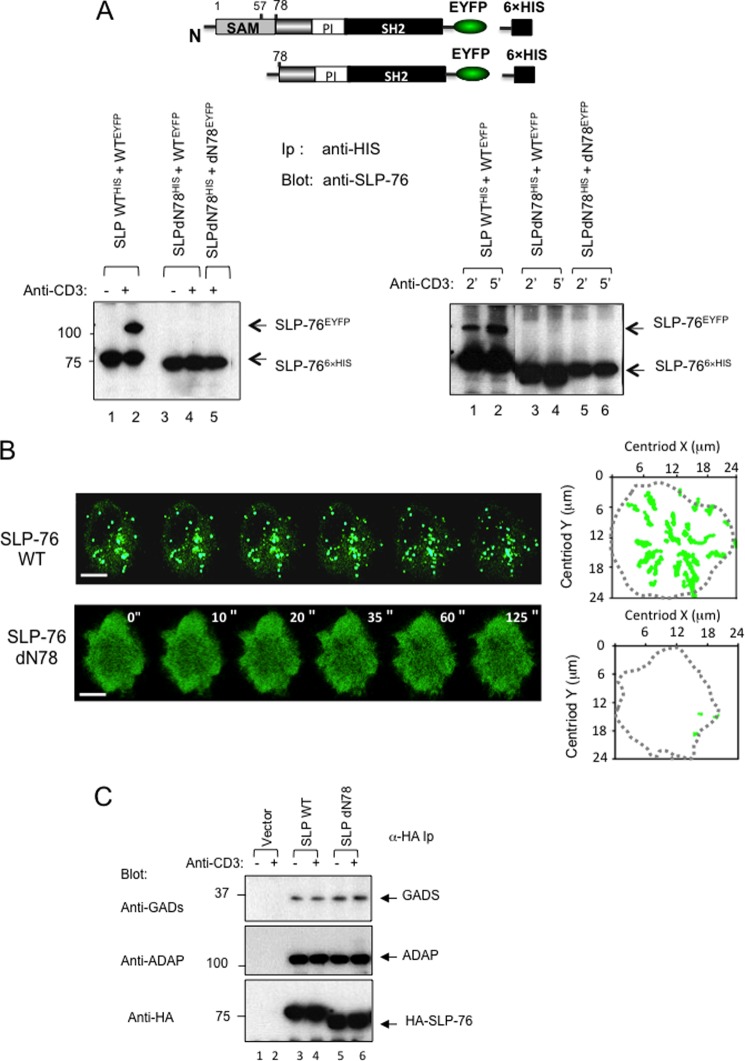
**Complete loss of the SAM region eliminates anti-CD3 induced SLP-76 dimerization and microcluster formation.** *A*, *upper panel*: schematic drawing of the C-terminally EYFP- or His-tagged SLP-76 WT and SAM domain deletion mutant dN78 constructs. *Lower panels*: deletion of the SAM region prevents SLP-76 co-precipitation of SLP-76. J14 T-cells were co-transfected and subjected to precipitation as described in Fig. 1 (*n* = 3). *Lower left panel*: SLPWT^His^ and WT^EYFP^ (*lanes 1* and *2*); SLPdN78^His^ and WT^EYFP^ (*lanes 3* and *4*); SLPdN78^His^ and dN78^EYFP^ (*lane 5*). *Lanes 1* and *3*, isotype IgG control; *lanes 2*, *4*, and *5*, anti-CD3. The *arrows* indicate the EYFP-SLP-76 (*higher band*) and His_6_-SLP-76 or His_6_-SLPdN78 (*lower band*). *Lower right panel*: anti-CD3 time course of ligation for 2 and 5 min as was indicated. SLPWT^His^ and WT^EYFP^ (*lanes 1* and *2*); SLPdN78^His^ and WT^YFP^ (*lanes 3* and *4*); SLPdN78^His^ and dN78^EYFP^ (*lanes 5* and *6*). *B*, time lapse images of SLP-76 WT and dN78 microclusters. J14 cells were transfected with SLP-76-EYFP WT (*upper panels*) or SLP-76-EYFP dN78 (*lower panel*). *Right panels*: tracking profiles of microclusters over time. The *dotted line* indicates boundary of T-cell/coverslip interface (*n* = 5). *Scale bar*, 10 μm. *C*, dN78 mutant retains binding to GADs and ADAP. The SLP-76 WT or dN78 mutant were transfected into J14 cells and subjected to immunoprecipitation with anti-HA antibody followed by blotting with GADS antibody (*upper panel*) and ADAP antibody (*middle panel*). Precipitated SLP-76 was verified by anti-SLP-76 blot (*lower panel*). *Lanes 1* and *2*, vector transfected; *lanes 3* and *4*, transfected SLP-76 WT; *lanes 5* and *6*, transfected SLPdN78. *Lanes 1*, *3*, and *5*, anti-IgG isotype control; *lanes 2*, *4*, and *6*, anti-CD3 ligation (*n* = 3).

Next, to assess whether SLP-76 could bind to itself in T-cells, two tagged versions of SLP-76 were generated, one with an YFP and another with a His_6_ tag (Fig. 2*A*, *left panel*). EYFP-tagged full-length SLP-76 (*i.e.* WT) was then co-expressed with His-tagged SLP-76 in SLP-76-deficient J14 Jurkat cells. Transfected cells were stimulated with anti-CD3 (*right panel*, *lanes 2* and *4*), or an isotype control (*lanes 1* and *3*) for 5 min, followed by lysis and precipitation of His-tagged SLP-76 with anti-His and blotting with anti-SLP-76. Significantly, anti-His precipitation of SLP-76-His co-precipitated SLP-76 EYFP from anti-CD3 ligated cells (Fig. 2*A*, *lane 4*). A weaker co-precipitated band was occasionally seen in resting cells (*lane 3*); however, in all experiments, the level of co-precipitation was markedly increased with anti-CD3 ligation. As a negative control, no SLP-76 was seen in the IgG control precipitates (Fig. 2*A*, *lanes 1* and *2*). SLP-76 binding to SLP-76 occurred as early as 2 min post-ligation and peaked at 5 min, followed by a slight decrease at 15 min post-anti-CD3 ligation (Fig. 2*B*, *lanes 2–4*). Concentrations of anti-CD3 as low as 0.5 μg/ml induced binding and this increased slightly with higher concentrations of 2 and 5 μg/ml (Fig. 1*C*, *lanes 2–4*). Importantly, the deletion of the SAM domain eliminated the ability of SLP-76 to co-precipitate SLP-76 (Fig. 3*A*). Although His-tagged WT SLP-76 co-precipitated EYFP-SLP-76 in response to anti-CD3 ligation (Fig. 3*A*, *left lower panel*, *lanes 1* and *2*), His-tagged dN78 (lacking residues 1–78) failed to co-precipitate WT EYFP-SLP-76 (*lanes 3* and *4*) or EYFP-dN78 (*lane 5*). No co-precipitation was seen at either 2 or 5 min post-anti-CD3 ligation (Fig. 3*A*, *right lower panel*, *lanes 3–6*) contrary to the wild type SAM domain (*lanes 1* and *2*). Consistent with SAM domain binding to itself to form higher order complexes, these observations showed that anti-CD3 induces self-association of SLP-76, which is dependent on the SAM domain.

SLP-76 forms microclusters in response to anti-CD3 ligation (35, 36). To assess this in the context of the SAM domain, J14 cells expressing EYFP-tagged WT or dN78 SLP-76 were imaged on anti-CD3-coated slides, as described (35, 37, 54). WT SLP-76 formed microclusters that migrated to the inner contact region over time (35, 54) (Fig. 3*B*, also *right panel*). By contrast, EYFP-tagged dN78 SLP-76 completely failed to generate microclusters and, instead, showed a diffuse pattern of membrane localization (Fig. 3*B*, *lower panel*). This occurred despite the fact that dN78 could still co-precipitate GADS and ADAP (Fig. 3*C*). This showed that the SAM domain was essential for microcluster formation, to a greater extent than previously observed with the partial SAM domain mutant (49).

##### SAM H5 α Helix alone Can Support Self-association and Microcluster Formation

**FIGURE 4.**
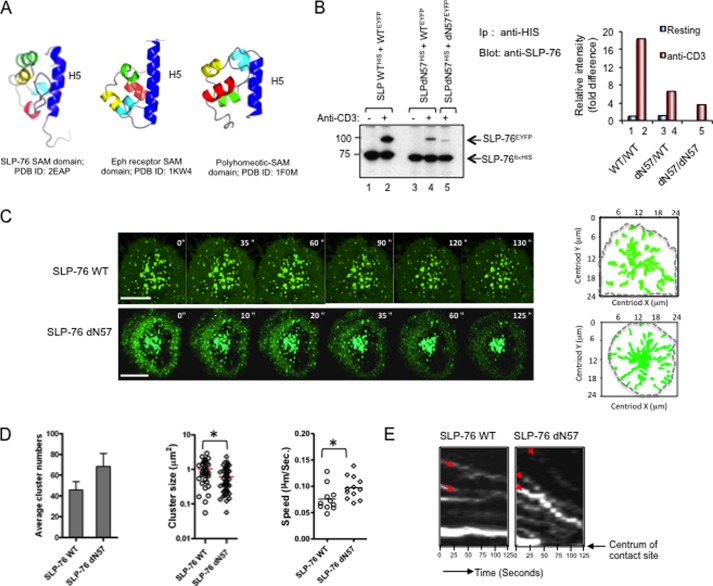
**SLP-76 dN57 with single SAM α helix supports binding to WT SLP-76 and dN57 and microcluster formation.** *A*, the ribbon structure of the SLP-76 SAM domain (Protein Data Bank (*PDB*) code 2EAP)^4^ relative to other solved Eph receptor and polyhomeotic SAM domain structures. Although the orientation of the individual N-terminal α H1–4 helices differs among SAM domains, the position of the H5 α helix is retained among the domains. With the ephrin receptor (EphB2) SAM domain, self-association is mediated by a hydrophobic interaction between amino-terminal H1–H4 helices and distinct binding between adjacent H5 helices and a nearby loop (44, 48). *B*, *left panel*: dN57 lacking H1–H4 domains but retaining H5 supports SLP-76 binding to itself. J14 T-cells were co-transfected with two tagged versions of SLP-76, one with an EYFP and another with a His_6_ tag and precipitated using anti-His monoclonal antibody as described in Fig. 1. *Lanes 1* and *3*, anti-IgG isotype control; *lanes 2*, *4*, and *5*, anti-CD3 ligation. *Lanes 1* and *2*, SLPWT^His^ and WT^EYFP^; *lanes 3* and *4*, SLPdN57^HIS^ and WT^EYFP^; *lane 5*, SLPdN57^His^ and dN57^EYFP^ (from same experiment as Fig. 3*A*, *left panel*). *Right panel*, histogram of relative intensities of band based on densitometric readings. *C*, tracking profiles of microclusters over time. The *dotted line* indicates boundary of T-cell/coverslip interface (*n* = 5). *Scale bar*, 10 μm. *D*, histograms showing the number of SLP-76-EYFP WT and SLP-76-EYFP dN57 clusters per cell (*left*), cluster sizes (*middle*), and speed of clusters (*right*) (*n* = 4). *Asterisks* indicate statistically significant *p* < 0.05 based on two-way analysis of variance. *E*, kymographs of movement of individual SLP-76-EYFP WT (*left*) and SLP-76-EYFP dN57 microclusters (*right*) over 125 s (*n* = 3). The *arrows* in *red* indicate the inward movement traces of individual SLP-76 microclusters (*n* = 3).

The crystal structure of the SLP-76 SAM domain has been solved recently^4^ (Protein Data Bank code 2EAP). The structure has similarities and differences to other solved SAM structures (42–45). The orientation of the individual N-terminal α H1–4 helices differs among SLP-76, EphB2 receptor, and polyhomeotic SAM domains, whereas the position of the larger H5 α helix is similar in each case (Fig. 4*A*). In this context, a previous study had shown that the self-association of the EphB2 SAM domain is mediated by two distinct interfaces: one by a hydrophobic interaction between amino-terminal H1–H4 helices and a second by the binding between adjacent H5 helices and a nearby loop (44, 48). This H5 interaction shows pseudodyadic symmetry in the packing of the monomer against the same region in another molecule (44).

To test whether the SLP-76 H5 domain could independently mediate SLP-76 self-association, the first four of the five SAM α helices (*i.e.* residues 1–57) were deleted leaving the single H5 helix attached to the rest of the SLP-76 protein (termed dN57). EYFP- and His-tagged versions were expressed in J14 cells, either alone or with WT SLP-76 (Fig. 4*B*). Remarkably, anti-His readily co-precipitated dN57^EYFP^ from lysates of cells co-transfected with dN57^HIS^ (Fig. 4*B*, *left panel*, *lane 5*). dN57 also co-precipitated SLP-76 WT^His^ when co-expressed in J14 cells (Fig. 4*B*, *lanes 3* and *4*). In both cases, the association was anti-CD3-dependent. As a further positive control, anti-His co-precipitated SLP-76 WT^EYFP^ from cells co-transfected with SLP-76 WT^His^ and SLP-76 WT^EYFP^ (Fig. 4*B*, *lane 2*). Densiometric readings showed that dN57 co-precipitated less dN57 than WT SLP-76 (*i.e.* 40% less). dN57 co-precipitation of WT SLP-76 was in turn less than WT SLP-76 co-precipitation of WT SLP-76 (*right* histogram). These observations showed that the SLP-76 SAM H5 helix was sufficient to bind an H5 helix in an adjacent SLP-76 molecule; however, this self-association was less efficient than to WT SLP-76 or between WT SLP-76 molecules with full-length SAM domains.

Intriguingly, dN57 SLP-76 also supported the formation of microclusters (Fig. 4, *C–E*). Furthermore, consistent with the reduced level of co-precipitated SLP-76, the dN57 microclusters were significantly smaller than SLP-76 WT clusters (Fig. 4*C*, *upper versus lower image*). Clusters form initially in the peripheral contact region followed by migration to the central contact region (35, 37, 54). Once the clusters migrated to the interior of the cell contact region, they coalesced to form larger clusters. Although the average size of the full length WT clusters in the peripheral contact region was 1.1 μm^2,^ the mean size of dN57 clusters was 0.62 μm^2.^ (Fig. 4*D*, *middle panel*). Interestingly, this reduced size was accompanied by an increase in the numbers of dN57 clusters (*i.e.* 70 clusters/cell to 47 for WT SLP-76 clusters) (*left panel*) and by a slight increase in the motility of clusters (0.096 *versus* 0.075 μm/s) (*right panel*). Kymograph analysis confirmed the more rapid movement of dN57 clusters (Fig. 4*E*). For 0–125 s, the dN57 clusters moved more rapidly to the central contact region than did the WT clusters. These data showed that the single SAM H5 helix self-association was sufficient to support SLP-76 microcluster formation.

##### SLP-76 SAM H5 α Helix Supports Enhanced T-cell Proliferation

**FIGURE 5.**
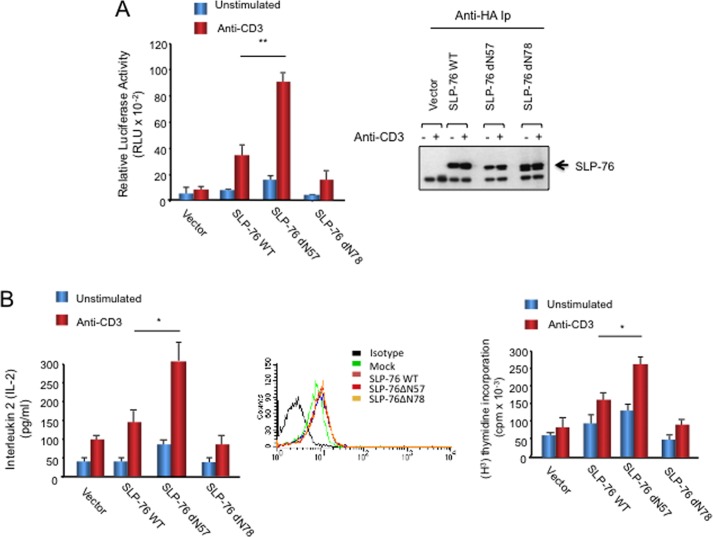
**dN57 supports anti-CD3-induced NF-AT promoter activity, IL-2 production, and proliferation of T-cells.** *A*, Jurkat J14 T-cells transfected with empty vector, HA-SLP-76 WT, HA-SLP-76 dN57 or HA-SLP-76 dN78 mutant, and luciferase-driven NF-AT promoter were stimulated with CD3 or isotype IgG control as indicated. *Right panel*: levels of transfected SLP-76 expression as detected by anti-SLP-76 blotting. *B*, dN57 supports anti-CD3 induced IL-2 production and proliferation in mouse and human primary T-cells. *Left panel*: mouse primary CD4-positive T-cells from spleen were stimulated with anti-CD3 for 24 h followed by a measurement of IL-2 production in supernatants using an ELISA assay. CD4^+^ cells were transfected with either control SRα vector, SLP-76 WT, SLP-76 dN57, or SLP-76 dN78 and incubated with anti-IgG isotype control (*blue bars*) or anti-CD3 (*red bars*) as indicated. Flow cytometric analysis of intracellular SLP-76 showed equal levels of transfected SLP-76 and its variants relative to endogenous SLP-76 in the mock control. *Right panel*: human primary T-cells were transfected with vector control, SLP-76 WT, SLP-76 dN57 or SLP-76 dN78 and incubated with anti-IgG isotype (*blue bars*) or anti-CD3 (*red bars*) for 36 h prior to incubation with [^3^H]thymidine for 12 h (*n* = 4). Similar levels of vector expression were obtained as seen in primary mouse cells.

dN57 SLP-76 also supported anti-CD3 induced NFAT-mediated transcription in J14 cells, as well as the production of IL-2 or proliferation of primary T-cells. Surprisingly, dN57 supported significantly higher levels of activation relative to WT SLP-76 (Fig. 5). J14 cells transfected with dN57 or WT SLP-76 with a 3×-NFAT-promoter construct were ligated with anti-CD3 followed by a measurement of luciferase activity. dN57 reconstituted the promoter activity at 2–3-fold higher levels than WT SLP-76 (*p* = 0.017). In contrast, dN78 SLP-76 failed to support increased transcription. As a control, the blotting of cell lysates confirmed expression of transfected SLP-76 constructs (Fig. 5*A*, *right panel*). Furthermore, the same effect of dN57 was observed in primary mouse and human primary T-cells (Fig. 5*B*). CD4-positive mouse T-cells were transfected and stimulated with anti-CD3 for 12 h followed by a measure of IL-2 by an ELISA assay (Fig. 5, *left panel*). Each transfected SLP-76 was expressed at similar levels (Fig. 5*B*, *middle panel*). dN57 enhanced IL-2 production relative to that seen with WT SLP-76, whereas the N78 mutant (a deletion mutant lacking N-terminal amino acids 1–78) failed to support IL-2 production. Similarly, transfection of primary human T-cells showed that dN57 greatly enhanced proliferation as measured by [^3^H]thymidine incorporation relative to WT SLP-76 and the dN78 mutant (Fig. 5*B*, *right panel*) (*p* = 0.043). Similar levels of expression of transfected constructs were observed in primary human and mouse cells. These data showed that the H5 SAM helix effectively supported the activation of T-cells.

**FIGURE 6.**
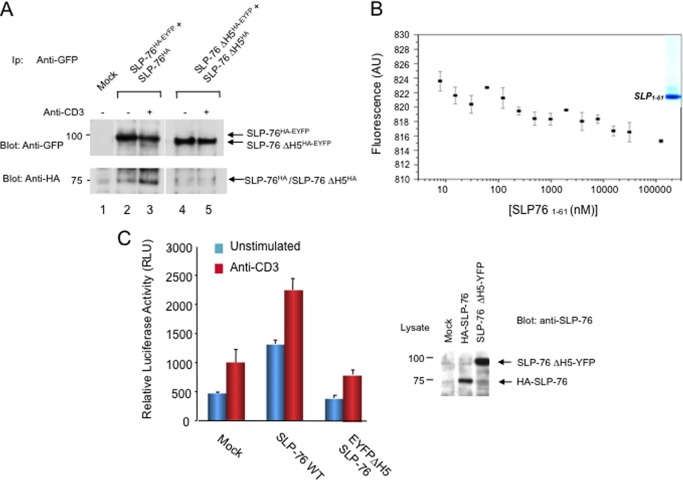
**Deletion of the SLP-76 H5 SAM subdomain impaired inter-binding and support of anti-CD3 induced NFAT-AP1 transcription.** *A*, deletion of the SLP-76 H5 SAM subdomain impaired interbinding. SLP-76-WT-EYFP (WT SLP-76^HA-EYFP^) was expressed with HA-SLP-76 (SLP-76^HA^) or SLP-76-ΔH5-EYFP (SLP-76ΔH5^HA-EYFP^) was expressed with HA-SLP-76-ΔH5 (SLP-76^HA^) followed by anti-CD3 ligation for 5 min and then precipitation with anti-GFP followed by blotting with anti-GFP (*upper panel*) or anti-HA (*lower panel*). *Lanes 2* and *4*, resting; *lanes 3* and *5*, anti-CD3-ligated cells. Shown is SRα vector expression alone (*Mock*) (*lane 1*), SLP-76-WT-EYFP expression with HA-SLP-76 (*lanes 2* and *3*) or SLP-76-ΔH5-EYFP expression with HA-SLP-76-ΔH5 (*lanes 4* and *5*) followed by anti-GFP precipitation and blotting for anti-GFP (*upper panel*) and anti-HA (*lower panel*) (*n* = 4). *B*, SLP-76 SAM lacking the H5 helix (residues 1–61) failed to show higher order complexes as monitored by MST. Analysis of SLP-76 protein residues 1–61 was analyzed by MST as described in Fig. 1 and under “Experimental Procedures.” *C*, SLP-76 ΔH5-EGFP failed to support anti-CD3-induced NF-AT/AP1 promoter activity in J14 Jurkat cells. J14 cells were co-transfected with SRα vector (mock), SLP-76 WT or SLP-76 ΔH5-EYFP plus a NF-AT/AP1 luciferase promoter. Eighteen hours after transfection, cells were stimulated with anti-CD3 for 6 h, followed by a measure of luciferase activity. *Right panel*: anti-SLP-76 blotting of lysates from transfected J14 cells.

To further assess the importance of the H5 motif in inter-SLP-76 binding, a deletion mutant form of SLP-76 lacking the H5 domain (ΔH5) was generated and expressed in J14 Jurkat T-cells **(**Fig. 6). SLP-76-WT-EYFP was expressed with HA-SLP-76 WT, or SLP-76-ΔH5-EYFP was expressed with HA-SLP-76-ΔH5 followed by anti-CD3 ligation for 5 min and then precipitation with anti-GFP followed by blotting with anti-GFP or anti-HA. As shown previously, anti-GFP precipitated SLP-76-WT-EYFP from resting and activated cells (Fig. 6*A*, *upper panel*, *lanes 1* and *2*) and co-precipitated HA-SLP-76 WT from stimulated cells (*lower panel*, *lane 3 versus 2*). Anti-GFP also precipitated SLP-76-ΔH5-EYFP from resting and activated cells (Fig. 6*A*, *upper panel*, *lanes 4* and *5*). However, it failed to co-precipitate HA-SLP-76-ΔH5 from resting or activated T-cells (Fig. 6*A*, *lower panel*, *lanes 4* and *5*). A faint amount of material in the HA-SLP-76-ΔH5 *M*_r_ range was occasionally seen; however, this observation was not reproducible. In agreement with the impaired self-association between SLP-76 lacking the H5 helix, we also examined *in vitro* self-association of the SAM domain lacking the H5 domain (*i.e.* SLP-76 residues 1–61) by MST (Fig. 6*B*). This assay showed that isolated protein fragment failed to show specific binding as seen by the absence of a sigmoidal shaped curve. Four different laser powers were used with a triplicate measurement at each power. Last, expression of the SLP-76-ΔH5-EYFP mutant failed to support an increase in anti-CD3-induced AP-1/NFAT transcription in T-cells (Fig. 6*C*). These data indicated that the H5 α helix is needed for the binding of the SAM domain to itself, and the ability of SLP-76 to generate signals from the T-cell receptor needed for NFAT-AP1 transcription.

## DISCUSSION

SLP-76 integrates signals from the antigen-receptor for the activation of T-cells. Despite this, it had been unclear whether the SLP-76 can directly self-assemble to form dimers and higher order oligomers in the generation of intracellular signals. Furthermore, although SLP-76 can form microclusters, it had been unclear whether self-assembly is needed for the formation of these large assemblies of proteins (35, 36, 50). In this work, we have shown for the first time that SLP-76 can self-associate in response to T-cell receptor ligation as mediated by its own N-terminal SAM domain. The purified SAM domain can form dimers, tetramers, and possibly higher ordered complexes, as detected by MST and DLS analysis. Furthermore, deletion of the SAM domain prevented SLP-76 self-association, whereas the retention of the single H5 helix sufficed to mediate self-association, albeit to a lesser degree than wild-type SLP-76. The H5 helix supported for formation of smaller clusters and enhanced T-cell activation. Overall, these observations show that SLP-76 SAM domain can self-associate for the formation of complexes for T-cell activation.

Our first observation was that the purified SLP-76 SAM domain self-associated to form dimers and other higher order oligomers as determined by MST and DLS analysis. We observed dimers as well as tetramers and possible higher order complexes. Dimer formation occurred at protein concentrations similar to, or lower than, those seen for other SAMs such in the EphB2 SAM domain (44, 48). The SLP-76 SAM domain therefore is a member of the minority of SAM domains (<25%) that have been reported or predicted to self-associate (41). We also observed that SLP-76 employed the SAM domain to self-associate in T-cells as seen by co-precipitation where anti-His co-precipitated SLP-76^EYFP^ with SLP-76^His^. This effect was lost with the dN78 mutant where the SAM domain has been deleted. Although some co-precipitation was occasionally observed in resting cells, the formation of the SAM-dependent complex was largely dependent on anti-CD3-induced signals. The nature of the T-cell receptor signals that facilitate this process is not known, but at a minimum, is likely to involve the increased plasma membrane localization for increased SLP-76-SLP-76 interactions. Despite the binding of SLP-76 to other proteins, GADs and ADAP, no SLP-76 co-precipitation of SLP-76 was noted with the loss of the SAM domain. This indicates that self-association depends on the SAM domain for assembly and cannot occur independently by the binding of other proteins to SLP-76. Instead, a more likely scenario would involve initial SAM mediated binding followed by the participation of other binding proteins, possibly for the assembly of even larger multiprotein complexes. Complexes involving two NCK and VAV1 molecules binding to SLP-76 have been described (50), whereas LAT can cluster independently due to the dimerization of GRB2 by SOS1 (61). SAM-mediated dimerization has also been shown to activate associated proteins such as in the ETS transcription factor TEL (TEL-SAM) (42). A similar activation event could possibly occur in the case of SLP-76 associated proteins.

Our second finding was that a subdomain of the SAM region, the C-terminal H5 helix alone, sufficed to support self-association. The observation was consistent with the conserved orientation of the H5 helix in different SAM domains, and the fact that the H5 helix in the EphB2 SAM domain serves as a second binding site between SAM domains (44). However, to our knowledge, the demonstration that the SLP-76 H5 helix alone can mediate SLP-76 dimer formation is the first example of autonomous SAM subregion mediated binding between SAM domains. At the same time, a major difference was seen in the efficiency of co-precipitation, where dN57 co-precipitated dN57 at lower levels than observed with full-length SLP-76. The importance of the H5 domain was further underscored by impaired ability of the intact SLP-76 protein with the deleted H5 helix (SLP-76ΔH5) to support co-precipitation in response to anti-CD3 ligation. MST analysis of the SAM domain lacking H5 helix also failed to show an evidence of self-association. Whether the H1-H4 region can form a second interface that depends on the presence of the H5 helix will require further structural analysis.

In the case of the dN57 *versus* WT SLP-76 proteins, confocal imaging showed remarkably that the H5 helix alone could support the formation of anti-CD3-induced clusters. This indicated that the observed H5 helix self-association was sufficient to mediate the formation of microclusters. However, the dN57 clusters were considerably smaller that the WT clusters as most evident in the peripheral region where clusters are known to arise. It is tempting to speculate that these smaller clusters represent dN57-dN57 dimers, whereas the larger WT clusters includes additional interactions that form larger oligomers. As well, upon migration to the interior of the cell contact region, the smaller dN57 clusters were observed to coalesce to form larger clusters, an event that we would speculate may involve the subsequent recruitment of other associated proteins such as VAV and NCK, which may also contribute to the formation of higher order complex structures. Although the complete loss of SAM completely prevented cluster formation, the partial deletion of residues 12–78 has been described to partially affect the longevity of cluster formation (49).

Last, the smaller sized oligomers and clusters of SLP-76 H5 supported IL-2 transcription in J14 cells at significantly higher levels than WT SLP-76 (Fig. 5). This effect was also seen in the activation of IL-2 production and proliferation in primary mouse and human T-cells. The basis for this gain-of-function is not clear but may involve the presence of a greater number and speed of microclusters for more interactions with other signaling proteins. Alternatively, although dimer formation is needed for the propagation of signals as shown by the loss of function with the dN78 and ΔH5 mutants, additional higher order complex formation may be inhibitory. Preliminary data showed that dN57 clusters have greater co-localization with LAT clusters (data not shown). The basis for the presence of a full-length SAM domain that limits the activation potential of the adaptor relative to the individual H5 helix remains to the uncovered. Further studies will be needed to uncover the role of individual components in the SAM region in the control of T-cell function.

## Footnotes

4 A. K. Goroncy, M. Sato, N. Tochio, S. Koshiba, S. Watanabe, T. Harada, T. Kigawa, and S. Yokoyama, submitted for publication.

## Footnotes

3 The abbreviations used are:

SLP-76: Src homology 2 (SH2) domain-containing leukocyte protein of 76 kDa
SAM: sterile α motif
ADAP: adhesion and degranulation promoting adapter protein
GADS: Grb2-related adaptor downstream of Shc
EphB: ephrin B
MST: fluorescence microscale therophoresis
DLS: dynamic light scattering
NFAT: nuclear factor of activated T-cells
EYFP: Enhanced yellow fluorescent protein.
